# Endosomal trafficking and related genetic underpinnings as a hub in Alzheimer's disease

**DOI:** 10.1002/jcp.30864

**Published:** 2022-08-22

**Authors:** Adriana Limone, Iolanda Veneruso, Valeria D'Argenio, Daniela Sarnataro

**Affiliations:** ^1^ Department of Molecular Medicine and Medical Biotechnology Federico II University Napoli Italy; ^2^ CEINGE‐Biotecnologie Avanzate Napoli Italy; ^3^ Department of Human Sciences and Quality of Life Promotion San Raffaele Open University Roma Italy

**Keywords:** Alzheimer's disease, amyloid‐beta, autophagy, endocytosis, endolysosomal network, RPSA

## Abstract

Genetic studies support the amyloid cascade as the leading hypothesis for the pathogenesis of Alzheimer's disease (AD). Although significant efforts have been made in untangling the amyloid and other pathological events in AD, ongoing interventions for AD have not been revealed efficacious for slowing down disease progression. Recent advances in the field of genetics have shed light on the etiology of AD, identifying numerous risk genes associated with late‐onset AD, including genes related to intracellular endosomal trafficking. Some of the bases for the development of AD may be explained by the recently emerging AD genetic “hubs,” which include the processing pathway of amyloid precursor protein and the endocytic pathway. The endosomal genetic hub may represent a common pathway through which many pathological effects can be mediated and novel, alternative biological targets could be identified for therapeutic interventions. The aim of this review is to focus on the genetic and biological aspects of the endosomal compartments related to AD progression. We report recent studies which describe how changes in endosomal genetics impact on functional events, such as the amyloidogenic and non‐amyloidogenic processing, degradative pathways, and the importance of receptors related to endocytic trafficking, including the 37/67 kDa laminin‐1 receptor ribosomal protein SA, and their implications for neurodegenerative diseases.

**Abbreviations**
AD: Alzheimer's disease
AICD: APP Intracellular Domain
APOE: apolipoprotein E
APP: amyloid precursor protein
APPL1: adaptor protein containing pleckstin homology domain, phosphotyrosine binding domain, and leucine zipper motif
Aβ: amyloid beta
BACE1: beta‐site APP‐cleaving enzyme 1; βCTF, beta C terminal fragment
BIN1: bridging integrator‐1
DS: Down syndrome
EOAD: early onset Alzheimer's disease
ER: endoplasmic reticulum
fAD: familial Alzheimer's disease
GVD: granulovacuolar degeneration
GWASs: genome‐wide association studies
LOAD: late onset Alzheimer's disease
LR: laminin receptor
LRP: density lipoprotein receptor‐related protein
PICALM: phosphatidylinositol binding clathrin assembly protein
PM: plasma membrane
PSEN1/2: Presenilin‐1 or ‐2
RPSA: ribosomal protein SA
SNP: single nucleotide polymorphism
SNXs: sorting nexins
sorLA: sortilin‐related receptor
TGN: trans Golgi network

## 1 INTRODUCTION

Alzheimer's disease (AD) represents the most common kind of dementia in the elderly (Blennow et al., 2006). Indeed, it is a progressive neurodegenerative disease featured by synaptic alterations and neuronal loss that lead to cognitive dysfunctions. Most of the AD cases are sporadic with a later in life onset (patients older than 65 years of age), and are usually referred to as late onset AD (LOAD) (D'Argenio & Sarnataro, 2020). LOAD is caused by a multifactorial pathogenesis due to a complex interaction of aging, genetics, environmental factors, and family history. However, rarer familial AD forms (fAD) have also been reported, in which clinical signs appear earlier during life (patients between 30 and 65 years of age). Hence, these cases are also referred to as early onset AD. The familial inherited forms of AD are due to rare causative mutations, and to date, amyloid precursor protein (APP), PSEN1, and PSEN2 have been identified as the three main involved genes, even if they explain just a small percentage of all fAD cases. The two main pathogenic features of AD brains are extracellular amyloid plaques and intraneuronal neurofibrillary tangles (Blennow et al., 2006). As a consequence, research on AD development and progression has been mainly focused on the understanding of the molecular mechanisms underpinning these two pathognomonic features, leading to the identification of amyloid beta (Aβ) peptides accumulation and tau fibrils hyperphosphorylation. Nevertheless, the underlining mechanisms through which these alterations promote AD are still not completely understood. Although senile Aβ plaques and neurofibrillary tangles are, to date, the most characterized neuropathological hallmarks of AD‐affected brain, several studies revealed enlarged endosomes and endolysosomal dysfunction as very early cytopathological signs within neurons of AD‐affected brain (Cataldo et al., 1997, 2000; Nixon & Cataldo, 1993). Thus, the involvement of the intracellular degradative pathways, that is, endocytic and autophagic route, has been proposed to contribute to alteration of protein homeostasis and the accumulation of misfolded proteins in AD (Cataldo et al., 1997; Gao et al., 2018; Jansen et al., 2019; Yu et al., 2005). Notably, these pathways are particularly relevant in neuronal cells as a protective factor against the accumulation of toxic compounds in physiological conditions. Based on this statement, it is quite obvious that alterations affecting these processes can be involved in several neurodegenerative diseases, including AD. In particular, the dysfunction of the endolysosomal trafficking, as well as of the autophagic process, is sustained by the observation of enlarged endosomes and the accumulation of autophagic vacuoles in AD‐affected brain. In support of this evidence, genome‐wide association studies (GWASs) identified trafficking machinery‐linked genes as risk factors for AD, highlighting the relationship between defects in membrane trafficking and Aβ production (Bellenguez et al., 2022; Gao et al., 2018; Jansen et al., 2019; Naj et al., 2014; Schwartzentruber et al., 2021). Collectively, the dysfunction of intracellular degradative pathways, the intracellular accumulation of Aβ peptides, together with other emerging molecular mechanisms, may contribute to cellular toxicity, and neuronal death. To date, it is clear that AD is an extremely complex condition determined by multiple, simultaneous and interacting pathophysiological processes and it is not possible to reduce its development to a single hypothesis. A better understanding of AD pathogenetic mechanisms will allow the development of novel targeted therapeutic approaches and better patient management. Here, we aim to review the current knowledge regarding the role of endosomal network alterations in AD, its interplay with amyloidogenesis, and the possibility to control Aβ generation and internalization by modulating the 37/67 kDa non‐integrin laminin receptor ribosomal protein SA (RPSA).

## 2 ENDOLYSOSOMAL NETWORK AND AUTOPHAGY DEFECTS OF ADAFFECTED HUMAN BRAIN

Beside the well‐established AD pathogenetic features, that is, Aβ peptides aggregation and tau protein hyperphosphorylation, an increasing body of evidence is highlighting the contribution of alterations of both the endolysosomal network and autophagic process in AD development (Cataldo et al., 1997; Jansen et al., 2019; Yu et al., 2005). Indeed, alterations in these pathways can be highlighted in the neurons of AD‐affected brains. The analysis of postmortem brains of AD patients revealed the presence of abnormally enlarged early endosomes in neurons within vulnerable regions of brain (individual endosomes displayed up to 32‐fold larger volumes than the normal average) (Cataldo et al., 1997), even in the absence of extracellular Aβ deposits and neurofibrillary tangles, that is, during earlier stage of the disease (Cataldo et al., 2000), thus suggesting a potential role in disease's progression. Moreover, the inheritance of the AD‐related risk factor Apolipoprotein‐E ε4 allele (*APOE* ε4) in heterozygosis, as well as in homozygosis, strongly accelerates the appearance of endosome abnormalities in earlier stages of the disease. Further evidence reinforcing the importance of endosomal dysfunction in AD pathogenesis derives from studies on section of prefrontal cortex from fetal, infant and young Down syndrome (DS) patients (typically developing AD), who already display early endosomes of abnormally large size. Accordingly, morphological and functional endocytic abnormalities in DS fibroblasts can be reversed by downregulating the expression of APP or BACE1, whereas, on the contrary, endosomal pathology can be induced in euploid fibroblasts by overexpressing APP or βCTFs (Jiang et al., 2010). Lately, enlarged endosomes have also been observed also in induced pluripotent stem cell‐derived neurons generated from sporadic LOAD patients (Israel et al., 2012) or from iPSC lines carrying *APP* and/or *PSEN1* mutations (Kwart et al., 2019), as well as in neurons or organoids derived from autosomal‐dominant AD cases (Raja et al., 2016). The enlargement of endosomal compartments is usually associated with both an accumulation of autophagic vacuoles (Nixon & Sheng Yang, 2011) and lysosomal impairment (Nixon & Cataldo, 1993). Accordingly, as reviewed in Nixon and Cataldo (1993), beside to alterations in the volume and size of early endosomal compartments, several studies on AD brains revealed a significant upregulation of lysosome activity and alterations in the localization pattern of acidic hydrolases. Both endocytic and autophagic systems transfer macromolecules to the lysosomes where they are degraded, hence a defective lysosomal clearance contributes to the accumulation of pathological protein aggregates in neurodegenerative disease, including AD. Indeed, mice with a brain‐specific deletion of autophagic and lysosomal regulators such ATG7 or TFEB, accumulate more Aβ (Nilsson et al., 2015; Reddy et al., 2016). However, to date genetic studies did not report lysosomal‐related gene variants directly associated with AD risk.

These lines of evidence, together with the absence of endosomes enlargement in other neurodegenerative diseases and in aging, as well as in advanced stages of familial form of AD caused by mutations in Presenilin‐1 or ‐2 (PSEN1/2) (Cataldo et al., 2000), strongly identified endolysosomal activation as a typical and early intracellular change occurring in sporadic LOAD, rather than a secondary effect of Aβ deposition.

The endocytic abnormalities found in neurons in AD patients are to date defined as one of the earliest cytopathological signs of AD and provide striking support for accelerated amyloidogenesis (Cataldo et al., 2000; Nixon, 2017).

## 3 ENDOCYTOSIS, β‐AMYLOIDOGENESIS AND PATHOGENESIS OF AD

Beside its role in the clearance of macromolecules, endolysosomal dysfunction is closely associated with amyloidogenesis, indeed APP normally traffics along the endocytic route where the production of Aβ peptide occurs (J. Z. A. Tan & Gleeson, 2019). Accordingly, we and others have demonstrated that intracellular trafficking is extremely important for the correct protein function and processing (Bhattacharya, Izzo, et al., 2020; Bhattacharya, Limone, et al., 2020; Sarnataro et al., 2017; J. Z. A. Tan & Gleeson, 2019). The intracellular trafficking of APP, as well as that of the secretases, plays a critical role in APP processing and in the production of Aβ; indeed, their co‐residence along trafficking routes, is crucial to understand where the processing of APP itself and the production of Aβ occurs.

APP is an integral type I membrane protein that is ubiquitously expressed in human tissues, and can be proteolytically processed along two alternative pathways: the non‐amyloidogenic and the amyloidogenic one (Zhang et al., 2011). In the non‐amyloidogenic pathway, APP undergoes the sequential cleavage by α‐ and γ‐ secretases, preventing the release of the neurotoxic Aβ peptide. In the amyloidogenic processing, APP undergoes the sequential cleavage by β‐ and γ‐secretases, thus leading to Aβ generation.

Once synthetized in the endoplasmic reticulum (ER), APP traffics along the secretory pathway and undergoes a series of posttranslational modifications, reaching the cell surface, from where it is endocytosed via a clathrin‐mediated endocytosis assisted by adaptor proteins AP‐2 and Dab2 (Mañucat‐Tan et al., 2019).

Beta‐site APP‐cleaving enzyme 1 (BACE1) is the main β‐secretase that initially cleaves APP to release Aβ (J. Z. A. Tan & Gleeson, 2019; Zhang et al., 2011). Once synthetized in the ER, BACE1 is transported across the Golgi apparatus to the cell surface, from where it is internalized into the endosomes. BACE1 is an aspartyl protease active at acidic pH; thus, its activity is exerted preferentially in acidic locations of the cell, such as trans Golgi network (TGN) and endosomes. Hence, both APP and BACE1 are found at the plasma membrane (PM); however, α‐secretase levels are higher at the PM and competes with BACE1 to process APP, thus most of the APP processing at the PM occurs via the non‐amyloidogenic pathway, while its processing along the amyloidogenic pathway occurs predominantly in intracellular compartments. From the PM both BACE1 and APP undergo endocytosis and traffic to the early endosomes: however, they are internalized through two different mechanisms. APP is mainly endocytosed via a clathrin‐dependent mechanism, while BACE1 via an ARF6‐regulated pathway (Sannerud et al., 2011). Secondary to β‐secretase, the γ‐secretase complex cleaves APP finally leading to Aβ release (Zhang et al., 2011). Although the catalytic subunit of γ‐secretase, PSEN1, has been found at the ER, Golgi, and post‐Golgi vesicles, the fully assembled γ‐secretase protease complex is generally found at the endolysosomal system (Pasternak et al., 2003).

Studies demonstrating a decrease in Aβ production by impairing acidification of endolysosomal system (Schrader‐Fischer & Paganetti, 1996), together with alteration of APP endocytosis under low‐potassium conditions (Koo & Squazzo, 1994), reinforce the scenario prospecting endosomes as a hub in AD pathogenesis.

Following the amyloidogenic cleavage of APP, Aβ peptides are secreted in the extracellular environment, where they can undergo to a self‐aggregation leading to the composition of Aβ fibrils and amyloid plaques (Zhang et al., 2011). This has been considered the main mechanism underpinning Aβ peptides pathogenicity in AD. Nevertheless, more recently, the focus has been moved to the possible toxicity related to the intraneuronal pool of Aβ peptides (Billings et al., 2005; Christensen et al., 2008; Knobloch et al., 2007). Studies on AD animal models have shown that intracellular accumulation of Aβ peptides is associated to synaptic function impairment, cognitive defects, and neuronal loss; moreover, these alterations may anticipate the external deposition of the amyloidogenic plaques (Billings et al., 2005; Christensen et al., 2008; Knobloch et al., 2007; Tomiyama et al., 2010). Interestingly, intracellular accumulation of Aβ peptides occurs within late endosomal multivesicular bodies and autophagic vacuoles, both showing also morphological alterations (Yu et al., 2005).

Beside their production within intracellular compartments, Aβ peptides exert toxic effects on the endolysosomal‐autophagic system itself, further amplifying its dysfunction and amyloidogenic processing (Almeida et al.,^2006^; Tammineni et al., 2017). Indeed, the accumulation of Aβ in endosomes contributes to their dysfunction, which in turn favors the amyloidogenic processing, thus promoting a cycle of self‐propelling endosomal dysfunction and excessive Aβ production (Schützmann et al., 2021). In this context, acidic vacuoles try to eliminate these toxic peptides: however, with the progressive increase of their concentration, they lose their physiological features and contribute to neurotoxicity. To note, the impairment of the endolysosomal‐autophagic system may be also related to other AD hallmarks, that is, tau hyperphosphorylation, by regulating the activity of enzymes involved in this process (Inoue et al., 2012; Platta & Stenmark, 2011). Several enzymes have been involved in tau phosphorylation and, since it has been reported that endosomal, and to a lesser extent, autophagosomal membranes, exert a regulatory role, it has been hypothesized that alterations of these pathways may explain the altered activity of these enzymes, at least in part. Moreover, the autophagy deficit may also reduce the ability of neurons to eliminate hyperphosphorylated tau, contributing to its toxicity (Polito et al., 2014).

Thus, taken together, these data support the idea that the integrity of the endolysosomal‐autophagic system is crucial for neuronal cell functions and, consequently, its dysfunction may lead to the onset of pathologic conditions (Tammineni et al., 2017). Hence, interest in neuronal endolysosomal system and its interplay with amyloidogenesis has recently grown because of its genetic and functional implication in AD.

## 4 GENETIC AND FUNCTIONAL IMPLICATIONS FOR ENDOLYSOSOMAL DYSFUNCTION IN AD

We just discussed how endolysosomal and autophagic dysfunctions may contribute to the onset of the two major AD hallmarks. However, what are the molecular mechanisms underlying endolysosomal‐autophagic pathway impairment in AD neurons? Since the correct functioning of both the autophagic and endocytic systems is a complex and finely regulated machinery, several factors may be involved in their alterations. The endosomal structural anomalies of AD, defined as “endosomopathy of AD,” encompass functional and morphologic abnormalities of endosomes that derive mainly from the overactivation of the small GTPase Rab5 (Kim et al., 2016), as well as alterations of trafficking route to and from sorting endosomes (Small & Petsko, 2015). Rab5 overactivation on endosomes in AD, derives mainly from the interaction between the high level of APP‐βCTF and APPL1 (adaptor protein containing pleckstin homology domain, phosphotyrosine binding domain, and leucine zipper motif) that is recruited to the Rab5 complex on endosomes (Kim et al., 2016). APPL1 stabilizes Rab5 active GTP isoforms in the membranes of endosomes, amplifying Rab5 signaling that is deleterious to endosome motility, endolysosomal cargo processing (including APP), synaptic plasticity, and neurothropic support to cholinergic neurons (Nixon, 2017). Interestingly, among the factors responsible for alteration of the intracellular trafficking, DNA variants occurring in genes involved in these pathways, by modifying the activity of the coded proteins, may play a role in the disease's development. The most well‐established genetic alterations associated to β‐amyloidogenesis, are DNA variants occurring in the *APP, PSEN1, PSEN2*, and *APOE* genes (D'Argenio & Sarnataro, 2020). However, known mutations in these four genes account only for a small proportion of all the genetic risk associated to AD. GWASs are highlighting several genetic variants related to increased risk of AD, among which different endolysosomal‐related genes have been reported (Jansen et al., 2019). Several recent GWASs strongly revealed endolysosomal genes to be enriched for genetic variants that convey increased risk of Alzheimer's disease, giving striking support to the endolysosomal hypothesis of neurodegeneration (Bellenguez et al., 2022; Gao et al., 2018; Schwartzentruber et al., 2021). Notably, differently from other neurodegenerative diseases (including Parkinson's disease, amyotrophic lateral sclerosis, or frontotemporal dementia), for which risk variants in genes directly linked to lysosomal function and homeostasis have been identified (reviewed in Udayar et al., 2022), the vast majority of the factors genetically related to AD susceptibility is linked to endosomal trafficking, rather than degradative pathway and/or lysosomal function.

For instance, among the genes related to the risk of LOAD developing, adaptor proteins, such as the X11‐family proteins, Fe65, LRP (density lipoprotein receptor‐related protein), sorting nexins (SNX), phosphatidylinositol binding clathrin assembly protein (PICALM) (Merthan et al., 2019) and sortilin sorLA (Yang et al., 2013), have been recognized to bind AICD (APP Intracellular Domain) and regulate APP trafficking and processing (Müller et al., 2008). The APP tyrosine‐based motif YTSI, located at its cytoplasmic tail, has been revealed to be involved in mediating endocytosis and processing (Tam et al., 2016).

### 4.1 X11‐family proteins

X11‐family proteins, interacting with APP, promotes its non‐amyloidogenic processing. Indeed, X11α knockout, X11L knockout, X11α/X11L double knockout AD mouse models showed an increased Aβ production (Kondo et al., 2010). The endocytic APP sorting process is regulated by Src‐mediated phosphorylation of Mint2/X11L that accelerates APP endocytosis and enhances APP sorting to autophagosomes, causing increased accumulation of Aβ (Chaufty et al., 2012).

### 4.2 Fe65

Fe65 is another adaptor protein interacting with AICD, its overexpression correlates with increased Aβ release, which is on the contrary reduced when Fe65 is knocked down (Bórquez & González‐Billault, 2012). Moreover, Fe65–APP complex acts as a transcriptional regulator by binding to other nuclear proteins including Tip60 (histone acetyl transferase Tat‐interacting protein 60 kDa). The genes regulated by Fe65–APP signaling include, among other less characterized genes, genes clearly involved in AD pathogenesis, such as glycogen synthase kinase‐3β (GSK3β), BACE1, neprilysin, and APP itself. Fe65 also interacts with LRP1, facilitating APP endocytic trafficking and modulating APP internalization increasing Aβ generation (Pietrzik et al., 2004).

### 4.3 SNX proteins

The α‐secretase cleavage of APP and the consequent reduction of Aβ production, has been reported to be favored by SNX33 (Schöbel et al., 2008). In particular, SNX33 binds to GTPase dynamin (protein involved in the fission of vesicles from donor membranes) impairing the rate of APP endocytosis in a dynamin‐dependent manner, with the consequent stabilization of APP on the membrane and its availability for α‐secretase cleavage. This effect is similar to the expression of the dominant negative dynamin‐1 mutant K44A. On the other hand, an increased APP trafficking to endosomes generating Aβ, has been described after overexpression of a dominant‐negative mutant of SNX17, as well as SNX17 knockdown (J. Lee et al., 2008).

### 4.4 PICALM

PICALM is an adaptor protein implicated in clathrin‐mediated endocytosis, indeed it binds to phosphatidylinositol 4,5‐bisphosphate (PIP2) to recruit AP2 and clathrin for receptor‐mediated endocytosis (Xiao et al., 2012). Hence, PICALM is involved in APP endocytosis and processing thereby influencing Aβ generation. In addition, Ando et al. (2013) demonstrated an aberrant processing of PICALM and a concomitant decrease in the expression of the full‐length isoform in AD brain samples. Moreover, PICALM is associated with neurofibrillary tangles in sporadic late onset AD, familial AD and DS cases, but not with amyloid plaques. PICALM's role in AD pathogenesis is further reinforced by genome‐wide association studies that identified SNPs a mechanism underlying the increased AD risk in *PICALM* variants' carriers (Harold et al., 2009; Kanatsu et al., 2016). For instance, the SNPs rs3851179 and rs541458 were found to be linked to LOAD (Harold et al., 2009). Interestingly, the SNP rs3851179 has been associated to thickening of entorhinal cortex and hippocampal degeneration (Biffi et al., 2010). Moreover, Morgen et al. (2014) showed a synergic effect of *PICALM* SNP rs3851179 and *APOε4* allele on cognitive decline and brain atrophy in AD. On the contrary, the SNP rs541458 in *PICALM* has been linked to decreased level of Aβ42 in CSF (Schjeide et al., 2011). Another protective variant (rs3851179) has been identified in *PICALM*, even if its effect seems to be related to *APOE* allelic status (Masri et al., 2019).

### 4.5 BIN1

Another protein that regulates membrane dynamics and that has been linked to AD pathology is Bridging Integrator‐1 (BIN1). Beside dynamin interaction, the neuronal isoform of BIN1 has been identified as an interactor of amphiphysin and, as a such, involved in clathrin‐mediated endocytosis (Ramjaun & McPherson, 1998). Loss of BIN1 in rodent neurons increased endocytosis and is associated with Rab5 positive endosome enlargement (Ubelmann et al., 2017). On the contrary, loss of BIN1 in human neurons is associated with decreased early endosomes size, whereas its overexpression caused an enlargement of early endosomes (Lambert et al., 2022). Although the inconsistency of these findings in rodents and human neurons, altogether they strongly reinforce the involvement of BIN1 in endocytosis regulation and AD pathogenesis. Moreover, it has been also shown that BIN1 depletion impacts BACE1 trafficking, causing its endosomal accumulation and, consequently, favoring Aβ production (Ubelmann et al., 2017). In line with this evidence, rare coding variants including rs754834233 or rs138047593 impact on AD risk reducing BACE1 recycling and causing early endosome enlargement recapitulating the same phenotype of Bin1 knockdown (Perdigão et al., 2021; Vardarajan, Ghani, et al., 2015). Beside this, BIN1 expression correlates also with tau pathology (Calafate et al., 2016), indeed it directly interacts with tau and such interaction is downregulated by tau phosphorylation on Thr231 (Sottejeau et al., 2015). Accordingly, BIN1 SNP rs59335482 is associated with an increased tau pathology, even if it does not influence Aβ accumulation, thus reinforcing the role of BIN1 in tau‐related AD pathogenesis (Chapuis et al., 2013). Moreover, Crotti et al. demonstrated that BIN1 overexpression promotes tau spreading via exosomes secretion (Crotti et al., 2019).

Secondary to APOE, BIN1 is the most important susceptibility locus for LOAD (Wijsman et al., 2011). To date, several large GWASs linked BIN1 variants to LOAD with high reproducibility (Harold et al., 2009; Jansen et al., 2019; Wijsman et al., 2011). The main associated SNPs have been identified in the 5' region of the gene, including the most significant SNPs rs744373 and rs7561528. Experimental data have shown that the BIN1 SNP rs7561528 associates with entorhinal and temporal pole cortexes thickness (Biffi et al., 2010), while the BIN1 rs744373 SNP correlates with the rate of cognitive decline and AD progression (Schmidt et al., 2012). Interestingly, the variant rs12989701, localized in the upstream region of the BIN1 locus in an evolutionarily conserved region, might be important for gene regulation.

Variants in BIN1 were significantly associated with LOAD also in Han Chinese individuals. Indeed, Tan et al. identified 44 variants in BIN1 gene including 2 rare missense mutations: P318L in exon 11 and P431L in exon 15 (M. S. Tan et al., 2014). Notably, only P318L showed significant association to LOAD risk, and it is predicted to have a harmful effect on BIN1 protein structure and function. Beside these missense mutations in BIN1 gene, Tan and colleagues also identified the minor G allele of the rs67327804 (G/A) polymorphism in intron 5 of BIN1 gene, to be associated with an increased risk of LOAD.

### 4.6 Retromer complex and sorLA/LR11

The retromer complex, which includes both a VPS35/26/29 trimer and a hetero/homodimer of sorting nexins (SNXs), known to be involved in the sorting from endosomes to the cell membrane or to the TGN, plays a pivotal role in intracellular trafficking and sorting regulation (Seaman, 2012). Retromer sorting has been implicated in Aβ peptides intracellular trafficking (Small & Petsko, 2015). A reduced expression of both VPS35 and VPS26 were reported in AD brain (Small et al., 2005), together with a higher Aβ deposition in retromer‐deficient animal models (Muhammad et al., 2008), highlighting the importance of cargo recycling from endosomes in the pathogenesis of AD. However, a recent study demonstrated that neurons of trans‐entorhinal cortex of the human brain, which is an AD‐vulnerable region, express high level of Vps26b, which is included in an alternative retromer complex responsible for endosomal recycling (Simoes et al., 2021). The current hypothesis is that a malfunctioning retromer may impair APP and BACE1 intracellular trafficking inducing their sequestration in the endosomes, thus leading to amyloid production.

Similar to the retromer complex, the type‐I membrane protein sortilin‐related receptor sorLA/LR11 (encoded by the *SORL1* gene) mediating APP trafficking via retromer association has been reported to be dysfunctional in AD (Wiinow & Andersen, 2013). SorLA is an adaptor molecule for retromers, indeed its cytoplasmic domain is recognized by the VPS26 subunit, thus controlling trafficking and amyloidogenic processing of APP (Fjorback et al., 2012). APP binding with sorLA facilitates APP trafficking from the plasma membrane to recycling endosomes allowing the sorting of APP in the TGN, reducing its amyloidogenic processing (Andersen et al., 2005). A reduced expression of sorLA has been reported in neurons of sporadic AD brain (Dodson et al., 2006); moreover, manipulating the expression of sorLA in non‐neuronal and neuronal cells, directly impacts APP processing (Young et al., 2015). Accordingly, neurons lacking sorLA expression show early endosomes enlargement and altered APP localization within the endo‐lysosomal compartments (Knupp et al., 2020; Mishra et al., 2022), thus reinforcing the involvement of sorLA in endosomal trafficking and APP processing. Besides its role in APP processing, sorLA also contributes to neurodegeneration because of its role in synaptic function, acting as a receptor for the neurotrophins BDNF and GDNF (Glerup et al., 2013; Rohe et al., 2013).

*SORL1* has been identified as an AD risk gene by genetic studies that identified several missense variants associated to AD susceptibility (Jansen et al., 2019; Rogaeva et al., 2007; Vardarajan, Zhang, et al., 2015). Among these, to note, the SNPs rs12285364 in *SORL1* was found to be significantly associated with an increased risk of AD developing. Moreover, exome sequencing studies have identified rare *SORL1* coding variants in patients affected by familial AD without known mutations in *APP* and *PSEN1/2* (Pottier et al., 2012). Although coding variants have also been identified in the control population, frameshift mutations responsible for premature stop codons in *SORL1* appear to occur only in AD cases (Pottier et al., 2012; Vardarajan, Zhang, et al., 2015).

### 4.7 CD2AP

Another AD‐risk gene is *CD2AP* which encodes for a protein involved in endocytosis and endosome's morphology (through interaction with Rab4) (Cormont et al., 2003; Gauthier et al., 2007), as well as in growth factor‐induced endocytosis (Kobayashi et al., 2004) and APP lysosomal trafficking (Furusawa et al., 2019). CD2AP is responsible for APP sorting to lysosome enhancing its degradation; accordingly, a reduced expression of CD2AP in neurons led to accumulation of APP in early endosomes, increasing amyloidogenic processing because of the increased convergence of APP and BACE1 (Ubelmann et al., 2017).

Several GWASs have identified *CD2AP* variants as AD risk factors (Hollingworth et al., 2011; Jansen et al., 2019). *CD2AP* rs9296559 and rs9349407 SNPs have been identified as AD susceptibility loci; however, the mechanism through which these variants may be related to AD has to be further investigated, even if it has been suggested a potential effect on blood‐brain barrier integrity and Aβ clearance (Cochran et al., 2015).

### 4.8 APOE

*APOE* gene encodes for a protein involved in lipid homeostasis and has been identified as the strongest risk factor for AD (Liu et al., 2013). The *APOE* gene exists as three polymorphic alleles, namely ε2, ε3, and ε4, which have a worldwide frequency of 8.4%, 77.9%, and 13.7%, respectively. These three ApoE isoforms differ in residues 112 and 158, where either cysteine or arginine can be present. The difference in amino acid composition at these positions affect the protein structure, as well as the ability to bind and transport lipid and Aβ. Individuals carrying ε4 allele in heterozygosis, as well as in homozygosis, have an increased risk of developing AD and lower age of onset compared to non‐carriers. On the contrary, the ε2 allele has a protective effect against AD. Beside its well‐established role in lipid homeostatis, ApoE has an important role in Aβ metabolism and its genotypes differentially influences Aβ deposition, aggregation, and clearance. Interestingly, the *APOE* ε4 allele correlates with Rab5 endosome enlargement (Cataldo et al., 2000). Indeed, the ApoE ε4 isoform has been related to a reduced endosomal recycling and increased endocytosis of both APP and BACE1 (He et al., 2007; Nixon, 2017). Moreover, in the presence of ApoE ε4 genotype, the level of BACE1 and βCTF, which specifically binds cholesterol (Barrett et al., 2012), increases causing over‐activation of Rab5 and consequent endosomal alterations (Cossec et al., 2010).

### 4.9 PSEN1

Among the proteins that display a crucial role in the functioning of endolysosomal and autophagic networks, a role for PSEN1 has been proposed. PSEN1 is the catalytic subunit of the γ‑secretase complex whose role has been well established in AD and also in familial AD (D'Argenio & Sarnataro, 2020). Beside this activity, PSEN1 plays a role in the endolysosomal‐autophagic system by regulating intracellular trafficking and autophagic degradation (J. H. Lee et al., 2010). Accordingly, deficient PSEN1 activity has been associated with the accumulation of autophagic vacuoles due to both lysosomal acidification failure and alteration of lysosomal calcium release. These mechanisms may be present not only in PSEN‐deficient familial AD, but also in LOAD. Interestingly, *PSEN1* deletion has been reported to increase lysosomal pH affecting intracellular trafficking of both endolysosomes and amphisomes (Catalog et al., 2004). Accordingly, *PSEN1* mutation, reducing protein activity, has similar effects on the endolysosomal compartment.

### 4.10 Granulovacular degeneration bodies

Another mechanism linking endolysosomal and autophagic pathway alterations to AD may be the presence of granulovacular degeneration (GVD) bodies. These latter are membrane‐bound intracellular organelles containing granules and autophagic‐like structures, whose accumulation has been proposed to be a consequence of an inefficient autolysosome production (Funk et al., 2011). Moreover, different tau kinases are associated with GVD bodies, thus further supporting the hypothesis that aberrant tau hyperphosphorylation may be a consequence of endolysosomal and autophagic pathway alterations (Yamazaki et al., 2011).

### 4.11 Rab10

Finally, in addition to genetic risk factors associated with an increased AD risk, GWAS has also identified some genetic variants in trafficking genes that seem to exert a protective effect (Jansen et al., 2019). For instance, a protective variant, rs142787485, identified in the 3′ untranslated region of *RAB10* has been associated with an AD risk reduction of 1.7 (Ridge et al., 2017). Since this variant reduces Rab10 expression, which is usually increased in AD, this has been hypothesized as a possible mechanism (Tavana et al., 2019). Moreover, Rab10 has been related to retromer function and other intracellular pathways, so that its role as neuroprotective factor may depend on different aspects.

## 5 THE ROLE OF 37/67 KDA LAMININ‐1 RECEPTOR (RPSA) IN THE INTERNALIZATION OF Aβ

Although several molecular mechanisms have been reported to be linked to Aβ‐mediated neuropathology, it seems that the intracellular trafficking and Aβ association with cellular receptors represent the key for understanding the intricate network underlying AD development. The internalization of Aβ oligomers through cell surface receptor correlates with AD progression, indeed once internalized, these oligomers cause cellular dysfunction and alteration of signaling pathway, thus contributing to the neuronal death (Stefani, 2010).

In the context of endocytic trafficking, the 37/67 kDa laminin‐1 receptor (LR, also known as RPSA) has recently been reported to mediate Aβ internalization and to be implicated in Aβ pathogenesis, as well (Da Costa Dias et al., 2014). Several studies have sustained the role of RPSA in the internalization of Aβ and Aβ‐mediated cytotoxicity, indeed anti‐RPSA specific antibody, as well as shRNA‐mediated downregulation of RPSA, rescues the viability and proliferative potential of cells treated with exogenous Aβ (Pinnock et al., 2016). Interestingly, this receptor has a high binding affinity for prion protein (PrP^C^), which in the context of AD, has been described to interact with Aβ oligomers and to mediate neurotoxic signals through Fyn kinase (Ji & Strittmatter, 2013). Whether RPSA directly mediates the internalization of Aβ, or whether it serves as the scaffold protein for the internalization of the PrP^C^‐Aβ complex, still remains an open issue.

Besides the role of RPSA in the internalization of Aβ, the receptor is also involved in the modulation of APP processing, further sustained by finding that the blockade of the receptor by means of antibodies, or its downregulation through shRNA, significantly reduces Aβ shedding (Jovanovic et al., 2013).

Likely, agents that induce RPSA endocytosis could have therapeutic potential against Alzheimer's disease and other AD‐related neurodegenerative diseases, such as Prion disease (Sarnataro, 2018; Sarnataro et al., 2016, 2017).

The use of receptor inhibitors revealed the possibility to modulate APP maturation and intracellular localization in neuronal cells, as well as to restore the lost “correct” APP intracellular localization, mitochondrial network and Aβ levels in fibroblasts from familial AD‐affected patients (Bhattacharya, Izzo, et al., 2020; Bhattacharya, Limone, et al., 2020).

**Figure 1:**
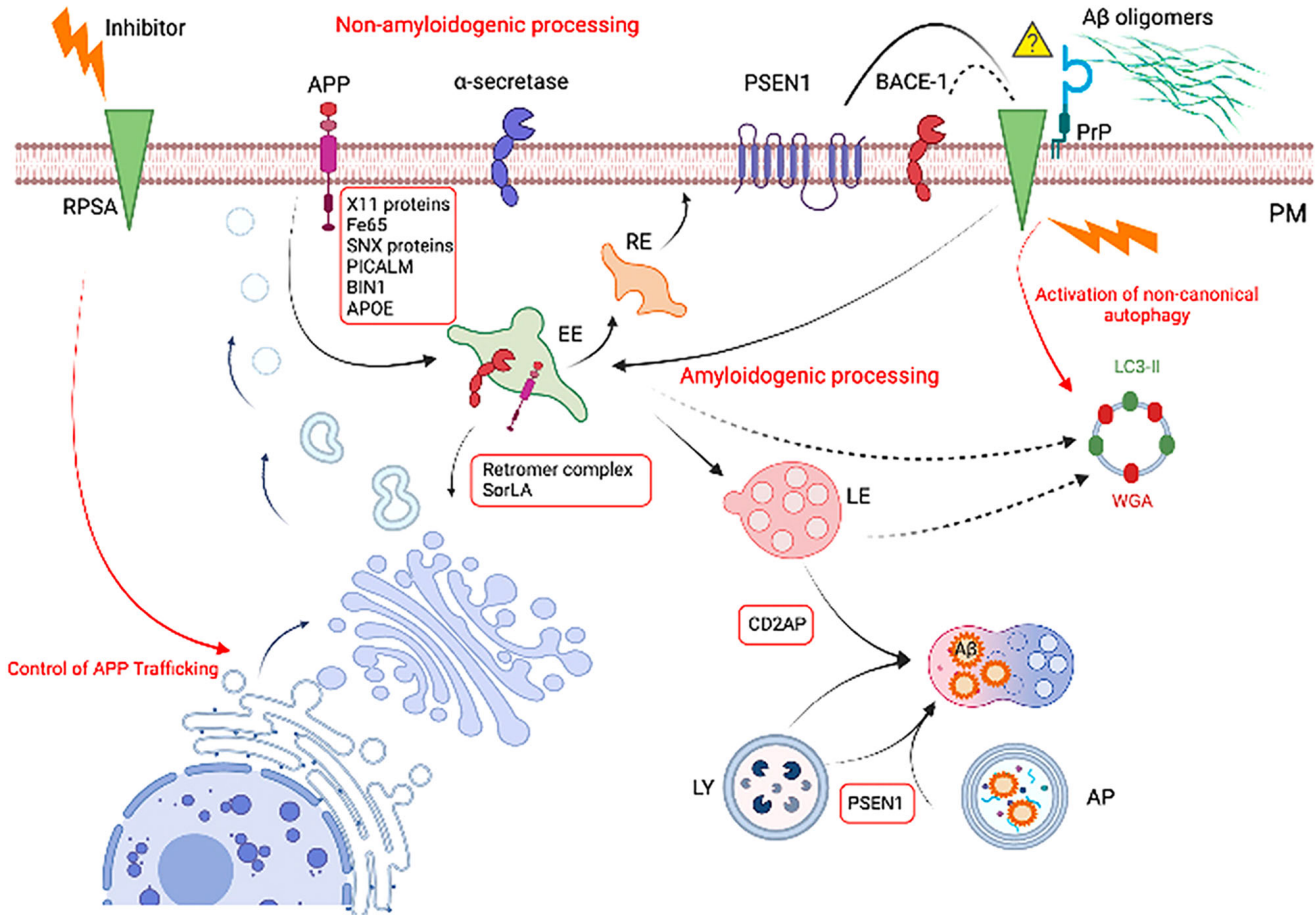
Schematic representation of the genes and pathways involved in AD pathogenesis. Intracellular trafficking and its regulation are particularly relevant for AD pathogenesis, indeed the trafficking of APP, as well as that of the secretases, plays a crucial role in amyloidogenesis. APP is synthetized in the endoplasmic reticulum and reaches the PM through the secretory pathway. At the PM, the prevalence α‐secretase favors the nonamyloidogenic processing of APP, whereas once internalized in the endocytic compartments, APP is mainly addressed by BACE1 to the amyloidogenic processing and the production of Aβ. The intracellular Aβ peptides accumulate within late endosomal multivesicular bodies and autophagic vacuoles (AP). Several factors regulating intracellular trafficking and sorting have been found to be dysfunctional in AD; among these, several gene variants have been identified to increase the risk of AD developing. Adaptor proteins, such as the X11‐family proteins, Fe65, SNX proteins, PICALM, APOE, and BIN1 regulate APP endocytosis, thus influencing its processing. Retromer complex and its adaptor protein sorLA control the recycling of APP from the endosomes to the TGN. CD2AP influences APP lysosomal trafficking. PSEN1 regulates lysosomal acidification. RPSA has been identified as one of the cell surface receptors responsible for Aβ internalization, however to date it is still unclear whether they directly interact or whether the prion protein PrP^C^ acts as scaffold in such interaction. Moreover, RPSA directly interacts with the subunit PSEN1 of γ‐secretase and indirectly with β‐secretase. Small molecules acting as specific inhibitors of the receptor modulate RPSA internalization and its interaction with PrP^C^. In addition, the receptor inhibitors modulate APP maturation and intracellular trafficking in neuronal cells as well as in fibroblasts from familial AD‐affected patients. Finally, inhibition of RPSA receptor induces unconventional endosomal LC3 lipidation and activation of autophagic pathways. AD, Alzheimer's disease; AP, autophagosomes; APP, amyloid precursor protein; EE, early endosomes; LE, late endosomes; PM, plasma membrane; PrP, prion protein. Created in BioRender.com

In addition, our recent findings of unconventional endosomal LC3 lipidation and activation of autophagic pathway through inhibition of RPSA receptor (Limone et al., 2022), highlight the strict link between endosomal network and autophagy and the possibility to modulate this process by RPSA inhibition. In Figure 1, we schematically report the genes and pathways involved in AD pathogenesis.

## 6 CONCLUSION

As reviewed herein, several alterations in endolysosomal and autophagic pathways, that is, “endosomopathy” have been described in AD and molecular alterations in gene coding for key proteins involved in these pathways have been also associated to an increased AD risk, thus supporting their role in the disease's onset and development. Even if further studies are required to establish the complex mechanisms linking both endolysosomal and autophagic pathways to AD onset and progression, all these lines of evidence clearly state that these two pathways are strictly related and that their balance is required for neuronal protein homeostasis. An increasing number of factors is able to perturbate this equilibrium and may play a role in AD pathogenesis. Elucidating the role of these genetic variants, both neurotoxic and neuroprotective, will not only improve the understanding of AD molecular mechanisms but will provide novel insights for the development of novel targeted therapies.
